# Simultaneous Serous Cyst Adenoma and Ovarian Pregnancy in An Infertile Woman

**Published:** 2014-03-09

**Authors:** Mahbod Ebrahimi, Firoozeh Akbari Asbagh

**Keywords:** Infertility, Ovulation Induction, Ectopic Pregnancy, Ovarian Pregnancy, Metformin

## Abstract

Ovarian pregnancy is a rare form of extra uterine pregnancy. Serous cyst adenoma is a benign variant of epithelial cell tumors of ovary. The coexistence of a cyst adenoma with an ovarian pregnancy in the same ovary is extremely rare. Some studies suggested that infertility or ovulation-inducing drugs can be involved in increased risk of ovarian tumors and ovarian pregnancies. A 28-year-old infertile woman presented with a ruptured ovarian pregnancy following ovulation induction with metformin. She had a concurrent benign serous cyst adenoma in the same ovary. Resection of both ovarian pregnancy and tumoral mass were performed. The ovary was preserved. Removal of gestational tissue and preservation of the involved ovary are the best options for management of ovarian pregnancy in young patient. Although there is an association between infertility/ovulation inducting medications and ovarian gestation, their connections with serous cyst adenoma are undetermined.

## Introduction

Ovarian pregnancy occurs when a fertilized ovum implants in or on the ovary. This entity is a rare variant of extrauterine pregnancy and represents 1.1-3% of all ectopic pregnancies or one ovarian pregnancy per 7000-90000 live births (1-4)

Serous cyst adenoma is a cystic ovarian tumor containing serous fluid and solid- tissue component. This tumor is benign form, presenting as cystic unilocular or multilocular ovarian mass with thin wall and minimal papillary projections (5).

Polycystic ovarian syndrome (PCOS) is the most common endocrinopathy among subfertile women (6). The most prominent presenting characteristics are oligo/anovulation and hyperandrogenism (7). Currently, metformin, an insulin-sensitizing agent, was accepted as a favorable medication for ovulation induction in PCOS (8, 9).

Infertility and subfertility managements including induction of ovulation seem to be responsible causes in the occurrence of the ectopic pregnancies (10-12).

In some retrospective studies, an association has been found between fertility drugs use and ovarian neoplasia risk in infertile patients (13-15).

The case presented here is interesting in term of the rarity of ovarian pregnancy and coincidence with serous cyst ovarian tumor, and also occurring after ovulation induction by metformin.

## Case report

In January 2013, a 28-year-old primigravida woman with sever lower abdominal pain presented to the emergency room of our hospital (Tehran Women General Hospital, Tehran, Iran). She suffered from vaginal spotting and lower abdominal pain for 5-6 consecutive days. She revealed a history of primary infertility with 3 years duration. Because of clinical and paraclinical manifestations of polycystic ovarian syndrome, metformin (1500 mg/day) has been prescribed for induction of ovulation since 8 months ago. After starting this medication, she developed regular menstruation pattern. Her last menstrual period was 25 days prior to admission date. She had no previous history of pelvic inflammatory disease, abdominal surgery, abortion, or use of any intrauterine contraceptive device (IUCD). Her hysterosalpingography (HSG) demonstrated an otherwise normal image without uteroovarian fistula. On general examination, she was pale. Abdominal examination showed abdominal distention and guarding. On vaginal examination, the uterus was normal in size and the cervix was tender in motion. There was a tenderous mass in deep palpation of right fornix. Clinical investigation showed hematocrit level of 25.2%, and betahuman chorionic gonadotropin (β-hCG) titer of 3569 m IU/mL. Vaginal ultrasonography demonstrated empty uterus with 6 mm endometrial thickness, free fluid in the peritoneal cavity, and a right sided heterogeneous adnexal mass (52×61 mm) beside the uterus. These findings were suggestive of ruptured ectopic gestation. Based on the above findings, the patient underwent emergency laparotomy in which demonstrated an enlarged and bluish right ovary with a 4 cm hemorrhagic and ruptured ovarian mass and a leaking hematoma on its surface. A 3×3 cm multicystic structure was identified in the other side of right ovary and was presumed to be a tumorous lesion. The uterus, both tubes and the left ovary appeared to be normal in appearance. The right tube had normal fimbriated end without dilation. There was no obvious evidence of endometriosis, metastatic lesions, pelvic inflammation, or adhesion. We found 1500 mL bloody fluid in abdominal cavity. The diagnosis of ovarian pregnancy was made. Therefore, surgical resection of hemorrhagic mass with conservation of the right ovary was done carefully. Because of bad looking appearance of the concurrent cyst in the right ovary, ovarian cystectomy and endometrial curetting were performed, respectively. The final pathologic analysis revealed vascularized chorionic villi and trophoblastic cells within ovarian paranchymal tissue (Figes1, 2). Histopathological study demonstrated that the excised cyst was a benign serous cyst adenoma (Fig 3). The endometrial sample showed decidual change, but no gestational tissue. The post-operative course was uneventful. On monitoring of β-hCG lev- els, they were undetectable (<5 m IU/ mL) on the 21^st^ postoperative day. The patient menstru- ated 37 days after the surgical operation.

**Fig 1:**
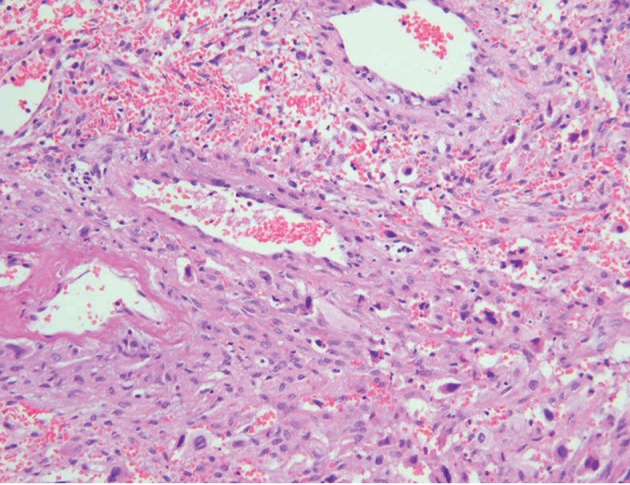
Photomicrograph identified trophoblastic cells within the ovarian parenchyma (H&E); ×200.

**Fig 2:**
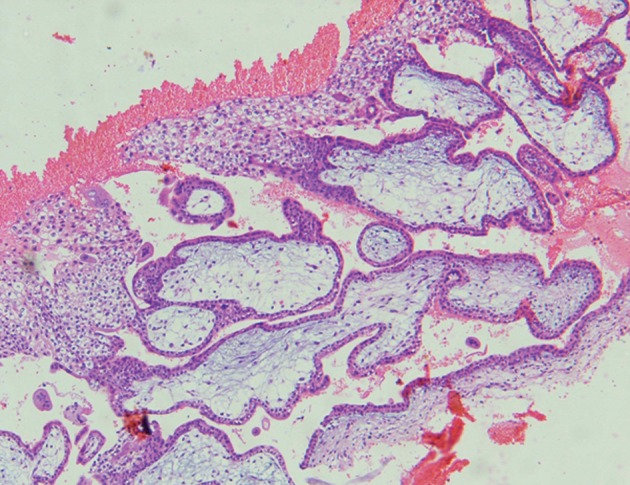
Photomicrograph identified chorionic villi (H&E); ×100.

**Fig 3:**
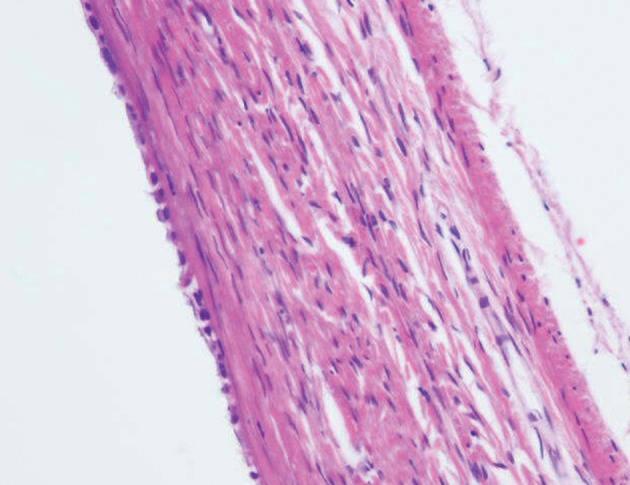
Photomicrograph identified serous cyst adenoma (H&E); ×400.

## Discussion

Ovarian pregnancy is a rare variant of ectopic pregnancy (1- 4). This entity must be documented by the four criteria of Spiegelberg: I. separation of intact tubes from the ovaries, II. a gestational sac occupying the normal position of the ovary, III. the ovary and sac connected to the uterus by the uteroovarian ligament, and IV. ovarian tissue histologically demonstrated in the sac wall (16).

 Its true incidence is underestimated. Some of the suspected tubal pregnancies that are approached with conservative managements, without laparoscopic validation, are in fact ovarian pregnancies (2, 10, 17). However, there is a rising in incidence of ovarian pregnancy in the last decade, its true incidence is undetermined (3, 10, 18). Furthermore, some studies suggested infer- tility and the medications, which are prescribed for ovulation induction, were associated with an increased incidence of ovarian pregnancies (3, 10-12). The suspected predisposing factors were increased levels of estrogen and progesterone after ovulation induction (19). However, the other reports did not support this hypothesis (2, 10, 20, 21)

 The exact etiologic factors of increased risk of ovarian pregnancy after ART programs are unclear, but the most likely mechanisms are as follows: reverse migration of embryo after deep deposition, the use of large volumes of culture fluid during embryo transfer (ET) procedures, difficult ET, and tubal pathologies (12, 22-24).

 The risk of ovarian pregnancy in patients with endometriosis or using IUCDs is controversial (3, 25). Unlike tubal pregnancy, the history of pelvic inflammatory disease does not increase the risk of ovarian pregnancy (2, 20, 21, 26).

Preoperative diagnosis is a challenge to the clinician due to its rarity and lack of typical presenting symptoms or documented risk factors (27). The clinical findings are similar to tubal pregnancy or hemorrhagic corpus luteal cyst (2, 28). The most complaints are abdominal pain and vaginal bleeding (3, 10). The increased vascularity of the ovary facilitates more massive bleeding and hypovalemic shock (3, 18). The asymptomatic patients are incidentally discovered during post- *in vitro* fertilization (IVF) monitoring (2). The ovarian pregnancies could be multiple gestations or heterotopic type (2, 29). There are very few recorded cases in which ovarian pregnancy reached viability (30).

The ultrasound features are ovarian enlargement with or without containing a double hyperechogenic ring along with yolk sac, fetal part, or fetal heart beat within ovary; fluid collection surrounding the ovary; and an empty uterus (2, 4, 20, 31, 32). The sonographic differential diagnosis between ovarian pregnancy and a ruptured corpus luteal cyst or a hemorrhagic ovarian tumor is difficult (2, 4, 27, 33). Although definite diagnosis is reached by laparoscopy or laparotomy, some of ovarian pregnancies are identifiable by vaginal ultrasound scanning (10, 17). Ovarian pregnancies can be mistaken for hemorrhagic corpus luteal cysts or pregnancies in the distal part of tube even at laparoscopy or laparotomy (3). Since pregnancy induced tissue destruction and lesser tissue is available in conservative surgeries, postoperative diagnosis is also difficult (21). Therefore, the physicians must exhibit a high degree of suspicion for ovarian pregnancy when managing with pregnancy of unknown location.

In recent years, accurate and earlier diagnosis has been performed by the application of vaginal ultrasound scanning and quantitative hCG measurement (1, 28). So, the management procedures of ovarian pregnancy have evolved oopherectomy by open surgical procedures and removal of gestational products or ovarian wedge resection by the laparoscopy and/or medical management using intramuscular or local injection of chemotherapeutic agents such as methotrexate (50 mg/m2) , hyperosmolar glucose, and prostaglandins, especially in young patients who have an intact ovarian pregnancy and a desire for future childbearing (2, 3, 10, 17, 20, 29, 34-36). Application of these modalities in management of ovarian pregnancy decreases maternal mortality and morbidity rates (34).

Serous cyst adenoma is the most common benign epithelial cell tumor of ovary (5). In some retrospective epidemiologic studies, an association has been demonstrated between prolonged infertility/use of ovulation inducing drugs and an increased incidence of ovarian epithelial cell dysplasia and cancer (13-15, 37-40). The stimulation of ovulation and increased levels of estrogen and progesterone explain the potential suspected relationship between the use of fertility medications and development of ovarian neoplasia (39)

In the other hand, a high frequency of hyperplasia and metaplasia in the ovarian epithelial surface and 2.5-fold increased risk of ovarian cancer have been showed in women with PCOS (41, 42). Although the contradictory data were reported in the other studies (40, 43-45, 46), like in a study by Brinton, the dosage and number of cycles of ovulation-inducing drugs were not associated with elevated risk (15). This difference is based on different study design, confusing factors such as duration and causes of infertility, parity, as well as type and duration of medical therapy (33).

In some cohort studies, ovulation-inducing drugs were related to an elevated risk of border line serous tumors (13, 40, 47). However, there is a possible association between prolonged use of clomiphene citrate and invasive epithelial ovarian tumors (40). This relationship should be pointed out with caution. The confounding influence of infertility and nulliparity should be kept in mind. Additionally, the other authors did not confirm these data in case-control studies (13, 15, 42, 48, 49).

Fertility experts frequently use metformin, an insulin-sensitizing agent, as an ovulation-inducing medication in PCOS (50, 51). Although its connection with ectopic gestation is reported (52), its association with serous cyst adenoma is undetermined. Some studies showed metformin use has been associated with a significant decrease in risk of prostate, pancreas, and breast cancer (53, 54); however, its molecular effect on ovarian tissue, and also, its effect on the risk of ovarian tumors remain undetermined (50). Although superovulation and increased serum levels of ovarian steroid hormones are the suspected pathogenesis of raising incidence of epithelial cell tumors following induction ovulation, superovulation is very rare event after metformin use (51); furthermore, estrdiol and progesterone serum levels are near the physiologic levels in cycles after being induced by this agent (50).

However, to the best of our knowledge, this study is the first case report of coexistence of an ovarian pregnancy and a serous cyst adenoma in the same ovary following ovulation induction with metformin. Review of the literature showed only a few reports presenting the coincidence of a serous cyst adenoma with an ectopic pregnancy. They have been cases involving tubal or abdominal pregnancy coinciding with serous cyst adenoma (52, 55, 56). Vazquez et al. (55) reported tubal pregnancy and ovarian serous cyst adenoma in a 40-year-old patient. The pregnancy was happened after induction of ovulation by clomiphene citrate. Pricop et al. (56) presented a case with abdominal pregnancy and serous cyst adenoma. Werlin et al. (52) reported a coincidence of tubal pregnancy with a serous cyst adenoma in the same fallopian tube following induction ovulation with metformin, letrozol, and low-dose gonadotropin. After review of literature, we did not find original study or documented case report about relationship between the use of metformin and ovarian tumors.

In our case, the presenting signs and symptoms were severe abdominal pain with vaginal spotting, elevated β-hCG, ovarian mass and empty uterus. She fulfilled the criteria for ovarian pregnancy, as by Spiegelberg’s outlines. She had a concurrent benign serous cyst adenoma in the same ovary. Our patient was previously labeled with PCOS. Pregnancy happened following induction of ovulation with metformin. In term of risk factors, infertility and the use of ovulation inducing drugs might be the possible predisposing factors for the ovarian pregnancy. The relationship between metformin and serous cyst adenoma is not clear. 

## Conclusion

Ovarian pregnancy is uncommon entity, which is difficult to diagnose. The microinvasive surgical procedures and medical managements are effective therapeutic options in the treatment of unruptured ovarian pregnancies, especially in young patients. Although the current findings are not strong to support a link between fertility drugs and ovarian cancer, it seems to be an association between reduced fertility and increased neoplasia risk. Careful inspection of the ovaries at surgery indicated the high risk of ovarian tumors for patients with long-standing history of infertility or fertility agents use in order to exclude the presence of a neoplasm. Moreover, further prospective, multicenter, and long follow-up studies considering all confounding factors are necessary to improve our ability for diagnosis and treatment of ovarian pregnancy, and to determine the patho-physiological mechanisms underlying the possible link between infertility or the use of ovulation inducing drugs and ovarian tumors. 
